# 

**Published:** 1853-05

**Authors:** 


### CONTENTS

OF NO. V.

### ORIGINAL COMMUNICATIONS.

ESSAYS AND CASES.

Art. I.—A Case of Penetrating Wound of the Chest; Pleuritis; Recovery. By James Knapp, M. D., of Louisville, Ky. . . . . . . . . . . . . 371

Art. II.—A Case of Extensive Abscess of the Liver; Effusion into the Peritoneum; Death; Autopsy. By J. R. Wilcox, M. D., of Evansville Ind. . . . . . . 375

Art. III.—A Case of Fatal Gangrene of the Fauces, caused or attended by Worms. By William Boyd, M. D., of Rock Island, Texas. . . . . . . . . 377

Art. IV.—A Case of Verminous Disease. By J. Windle, M. D., of Oskaloosa, Iowa. . . . . . 378

Art. V.—A Case of Typhoid Fever marked by some peculiarities. By William Kenney, M. D., of Millersburgh, Ky. . . . . . . . . . . . 379

Art. VI.—Quinine in Cholera Infantum. By G. W. Booth, M. D., of Carrolville, Miss. . . . . . . 384

Art. VII.—A Letter on the Physiological Doctrines of Marshall Hall, M. D. By B. M. Wible, M. D., of Louisville, Ky. . . . . . . . . . 385

REVIEWS.

Art. VIII.—What to Observe at the Bed-side and after Death in Medical Cases. Published under the authority of the London Medical Society of Observation. Philadelphia, Blanchard & Lea, 1853, 8vo., pp. 202. . . . 389

Art. IX.—Atlas of Pathological Histology. By Dr. Gottlieb Gluge, Professor of Physiology and Pathological Anatomy in the University of Bruxelles, Member of the Royal Academy of Bruxelles, &c. Translated from the German by Joseph Leidy, M. D., Pathologist to

St. Joseph's Hospital, Philadelphia, Fellow of the College of Physicians, Philadelphia, Honorary Fellow of the Medical Society of Virginia, Corresponding Member of the Biological Society of Paris, &c., &c. - 391
ART. X.—Report of the Eastern Lunatic Asylum in the city of Williamsburg, Virginia. 1852-3. - - - - 396
ART. XI.—A System of Practical Surgery, by WILLIAM FERGUSSON, F. R. S., Professor of Surgery in King's College, London; Surgeon to King's College Hospital; and Surgeon to H. R. H. Prince Albert. Fourth American from the third and enlarged London Edition, with three hundred and ninety-three illustrations. Philadelphia, Blanchard & Lea, 1853: 8vo., pp. 621. - - - 397
ART. XII.—Illustrations of Syphilitic Disease. By PHILIP RICORD. Translated from the French by THOMAS F. BETTON, M. D., M. A. N. S., Fellow of the College of Physicians of Philadeldhia, etc., etc. - - 411
ART. XIII.—Manual of Physiology. By WILLIAM SENHOUSE KIRKES, M. D., Licentiate of the Royal College of Physicians; Registrar, and Demonstrator of Morbid Anatomy, at St. Bartholomew's Hospital, etc., etc. - 413
ART. XIV.—The Druggist's General Receipt Book. By HENRY BEASLEY. 12mo., pp. 472. - - - - - 414
ART. XV.—Handbook of Natural Philosophy. By DIONYSIUS LARDNER, D. C. L., etc., etc. 12mo., pp. 451. - 415
ART. XVI.—Natural Theology. A Valedictary Lecture, delivered by JOHN LOCKE, M. D. - - - - - 415
ART. XVII.—On the Alteration of the Taste in Paralysis of the Facial Nerve. By M. CLAUDE BERNARE. - 417

SELECTIONS FROM FOREIGN AND HOME JOURNALS.

Case of Excision of the Scapula. . . . . . . . 419
Purging to prevent Traumatic Inflammation. - - - - 422
Origin of Epidemic Diseases. . . . . . . . . 424
Absence of Fibrin in the Blood. - - - - - - 428
Relations of Vaccination, &c., to Small-Pox. - - - - 429
Relations of Menstruation, Lactation, and Conception. - 432
Peculiar Case of Lithotomy; singular Nucleus for, &c. - 432
Use of Chloroform in Midwifery. - - - - - - - 435

ORIGINAL INTELLIGENCE.

American Medical Association. - - - - - - - 444
Accidental Death of Physicians. . . . . . . . 464

